# Socioeconomic position over the life course and impaired lung function of older adults in Central and Eastern Europe: the HAPIEE study

**DOI:** 10.1136/jech-2022-219348

**Published:** 2022-11-02

**Authors:** Consuelo Quispe-Haro, Andrzej Pająk, Abdonas Tamosiunas, Nadezda Capkova, Martin Bobak, Hynek Pikhart

**Affiliations:** 1 RECETOX, Faculty of Science, Masaryk University, Kotlarska 2, Brno, Czech Republic; 2 Department of Epidemiology and Population Sciences, Institute of Public Health, Jagiellonian University Medical College, Krakow, Poland; 3 Laboratory of Population Research, Institute of Cardiology, Lithuanian University of Health Sciences, Kaunas, Lithuania; 4 National Institute of Public Health, Prague, Czech Republic; 5 Research Department of Epidemiology and Public Health, University College London, London, UK

**Keywords:** life course epidemiology, health impact assessment, social class, health inequalities, epidemiology

## Abstract

**Background:**

Social differences in lung functioning have been reported, but the role of socioeconomic position (SEP) at different stages of life is less well understood, particularly in Central and Eastern Europe. This study addressed this question.

**Methods:**

The analysis included 10 160 individuals aged 45–70 years from the Czech Republic, Poland and Lithuania. Lung function was either normal if values of forced expiratory volume in the first second divided by forced vital capacity (FEV1/FVC) and FVC were higher than the lower limit of normality or impaired if otherwise. SEP at three stages of life was assessed using maternal education (childhood), participant’s education (young adulthood), and current ability to pay for food, clothes and bills (late adulthood). SEP measures were dichotomised as advantaged versus disadvantaged. The associations between impaired lung function and life-course SEP were estimated by logistic regression.

**Results:**

Disadvantaged SEP in young and late adulthood had higher odds of impaired lung function. In young adulthood, age-adjusted ORs were 1.26 (95% CI 1.06 to 1.49) in men and 1.56 (95% CI 1.29 to 1.88) in women, while in late adulthood, the ORs were 1.15 (95% CI 0.99 to 1.34) in men and 1.26 (95% CI 1.09 to 1.46) in women. Men and women disadvantaged at all three stages of life had ORs of 1.42 (95% CI 1.06 to 1.91) and 1.83 (95% CI 1.32 to 2.52), respectively, compared with those always advantaged. Smoking substantially attenuated the ORs in men but not in women.

**Conclusion:**

Reducing socioeconomic inequalities in young and late adulthood may contribute to reducing the risk of impaired lung function in late adulthood.

**WHAT IS ALREADY KNOWN ON THIS TOPIC**

- Previous studies reported that socioeconomic position (SEP) assessed at earlier stages of life was associated with a reduction in forced expiratory volume in the first second and forced vital capacity.

**WHAT THIS STUDY ADDS**

- Accumulation of disadvantaged SEP over the life course was associated with impaired lung function, mainly driven by disadvantages in young and late adulthood. Smoking appeared a part of the gradient in men but not in women.

**HOW THIS STUDY MIGHT AFFECT RESEARCH, PRACTICE OR POLICY**

- Interventions that aim to reduce inequalities at any stage in life have the potential to improve pulmonary health. More evidence is needed to confirm these findings, mainly from birth cohorts.

## Introduction

Disadvantaged socioeconomic position (SEP) has been linked to reduced lung function: a linear positive association between SEP and both forced expiratory volume in the first second (FEV_1_) and forced vital capacity (FVC) has been suggested by Hegewald and Crapo,^1^ and Rocha *et al*.^2^ A recent study demonstrated that ‘at age of 45 individuals in disadvantaged SEP had lost 4 to 5 years of healthy lung function when compared with advantaged SEP counterparts’,^3^ illustrating the magnitude of socioeconomic inequalities on pulmonary health. The issue of inequalities is particularly pertinent in Central and Eastern Europe (CEE). Populations in the region have had worse health status and lower life expectancy than Western Europe.^4–6^ The causes of high mortality and morbidity are complex but previous reports have documented strong effects of socioeconomic inequalities in the region on various health outcomes,^6–11^ including lung function.^12^

SEP is a social construct that includes, but is not limited to, education, income and occupation. The association between SEP and health can be explained through differential exposures to environmental or occupational exposures, acquisition of cognitive and non-cognitive skills, socialisation of health behaviours, the ability to purchase health services and the construction of social networks.^13^ A disadvantaged SEP could also be considered a cause of psychological and social stress, leading to the immune system’s disruption.^14 15^ In fact, disadvantaged SEP at any stage of life was associated with several health outcomes, including metabolic, cardiovascular and respiratory diseases.^16–18^

SEP is not static and can vary throughout life. Similarly, lung vulnerability to stressors changes with age.^19 20^ Several life-course models have been suggested to test the implications of temporal association between exposures and outcomes: the cumulative model considers any stage of life equally important with a dose–response effect; exposure to risk factors during a critical period model has irreversible consequences on health, while the consequences of exposure during the sensitive period can be reversible.^21^

Epidemiological studies which assessed the association between SEP and lung function focused on FEV_1_ and FVC as independent outcomes, but FEV_1_ and FVC are determined by individual height, sex, age and ethnicity. Furthermore, FEV_1_ and FVC peak at the age of 20 and progressively decrease with age.^22^ Standardised equations^23^ help to overcome these variabilities because they use height, sex, age and ethnicity to predict the normal lung volumes and capacities. They calculate the lower limit of normality (LLN) or fifth percentile that determines the threshold at which a given value of FEV_1_ or FVC is considered abnormal. However, for a diagnosis of lung impairment, a combination of these two indicators is needed because people could have a normal FEV_1_ in the presence of impaired lung function.^24^

This study aimed to assess the risk of impaired lung function of older adults by SEP at three time points in life. Life-course SEP models were assessed to differentiate between critical periods, sensitive periods, trajectories, social mobility and disadvantage accumulation.

## Methods

### Study design and participants

This study is part of the Health, Alcohol and Psychosocial factors In Eastern Europe (HAPIEE) study, details about the project are available elsewhere.^25^ This study used cross-sectional individual-level data from urban centres in three CEE countries (Czech Republic, Poland and Lithuania). Random samples of men and women aged 45–70 years at baseline in 2002–2005 were invited to participate. The overall response rate was 59%; non-respondents were more likely to be male, younger, less well educated and less healthy. Altogether, 26 568 individuals were recruited at baseline. A total 10 828 participants were excluded because they did not have spirometry data or did not meet reproducibility and repeatability criteria; 5386 had missing information on mother’s education, 90 in current economic circumstances, 45 in smoking status, 34 in height and 25 in participant’s education (online supplemental figure 1). Our analysis included 10 160 subjects with complete data. Information about sex, age, smoking status, mother’s education, participant’s education and current socioeconomic circumstances, was obtained using a standard questionnaire. Anthropometric measurements (including height) and spirometry were performed by trained personnel. All participants signed informed consent.

10.1136/jech-2022-219348.supp1 Supplementary data

### Lung function assessment

Micro-Medical Microplus spirometer was used to measure lung function. All participants performed at least three manoeuvres; the highest value of FVC and FEV_1_ was used if acceptable and reproducible criteria were met following the American Thoracic Society (ATS) and the European Respiratory Society (ERS) criteria.^26^ Exclusion criteria include participants with less than three manoeuvres and those who did not satisfy acceptability and reproducibility criteria.

Individual information on sex, age and height were used in standardised equations validated in non-smoking Caucasians.^23^ The output included the predicted values of the LLN for FEV_1_/FVC ratio and FVC in each participant. In the second step, the algorithm by Pellegrino *et al* 24 was adapted (online supplemental figure 2) to create a dichotomous outcome variable to define normal lung function (with values of forced expiratory volume in the first second divided by forced vital capacity (FEV_1_/FVC) and FVC equal or higher than the LLN) versus impaired lung function (FEV_1_/FVC or FVC lower than the LLN).

10.1136/jech-2022-219348.supp2 Supplementary data

### Socioeconomic position

SEP at three stages of life was estimated for all the participants: childhood, young adulthood and late adulthood. All three estimates of SEP were dichotomised as advantaged SEP (coded as 0 in tables) and disadvantaged SEP (coded as 1). Childhood SEP was based on retrospectively reported mother’s educational level: no education or primary education (1) and higher than primary education (0). Young adulthood SEP was based on the highest level of education accomplished by the participant: primary/vocational or secondary education (1) and higher than secondary education (0). The late adulthood SEP was constructed by combining three questions: ‘Do you have difficulties paying bills?’, ‘Do you have difficulties paying for clothes?’ and ‘Do you have difficulties paying for food?’ Those who never had any of the difficulties were classified as advantaged (0), while those who had had any difficulty were classified as disadvantaged (1).

### Statistical analysis

The associations between the outcome (impaired lung function) and the main exposures and further covariate (age, sex, smoking status and country) variables were assessed with multivariable-adjusted logistic regression models.

Based on the framework by Ben-Shlomo and Kuh,^21^ several life-course models proposed by Mishra *et al* 27 were tested to assess the relationship between impaired lung function with SEP. The covariates in all SEP models included age, sex and smoking status. All models were disaggregated by country. First, each SEP category was included one by one in the critical period model (model 1). For the sensitive period model (model 2), all SEP variables were included at once as independent variables (no interactions between them). With dichotomised SEP variables, eight different course life trajectories were tested in model 3. Individual’s social mobility, in model 4, was categorised as upward when a person started in a disadvantaged SEP in childhood and moved to an advantaged SEP during young adulthood and/or late adulthood; by contrast, for downward mobility, individuals had an advantaged SEP during childhood but disadvantaged SEP later in life. Finally, a cumulative score representing the sum of the times a person was in a disadvantaged SEP was used in model 5.

The associations between SEP and lung function impairment were estimated by logistic regression. Likelihood ratio tests were used to assess the heterogeneity in the associations by country and sex. Among all SEP variables, the smallest p values for interaction with country and sex were seen for the trend by disadvantage accumulation (p=0.123 and p=0.056, respectively). While this is not strong evidence for effect modification by either variable, the attenuation of ORs after adjustment for smoking (the most important confounder or mediator) was much more pronounced in men. Therefore, the main results are presented separately by sex. Statistical tests were performed using Stata V.16.1. A p value of <0.05 was considered statistically significant.

## Results

Of a total of n=10 160 participants (mean age=58.99 years), 4619 were men and 5541 women, with 8393 people classified as healthy, while 1767 (17.39%) were classified as having impaired lung function. Current and past smokers (smokers) represented 50.74% of our sample. The 78.82% of men with impaired lung function were smokers vs 46.61% of women with impaired lung function who were smokers (table 1). However, impaired lung function was higher among female non-smokers (53.39%) than male non-smokers (21.18%). Poland accounts for 49.04% of men and 48.53% of women with impaired lung function, while Lithuania had the lowest input with 17.67% men and 15.95% women.

**Table 1:** Characteristics of the participants (n=10 160)

	Healthy lung function		Impaired lung function*	
	Men n=3736	Women n=4657	Men n=883	Women n=884
Age, mean (SD)	59.16±7.44	58.59±7.39	60.05±7.10	59.38±7.23
Height (m), mean (SD)	1.73±0.06	1.60±0.06	1.72±0.06	1.59±0.06
Smokers, n (%)	2377 (63.62)	1671 (35.88)	696 (78.82)	412 (46.61)
Non-smokers, n (%)	1359 (36.38)	2986 (64.12)	187 (21.18)	472 (53.39)
Country				
Czech Republic, n (%)	1270 (33.99)	1742 (37.41)	294 (33.30)	314 (35.52)
Poland, n (%)	1453 (38.89)	1556 (33.41)	433 (49.04)	429 (48.53)
Lithuania, n (%)	1013 (27.11)	1359 (29.18)	156 (17.67)	141 (15.95)

*Sex-specific pulmonary function standardised for age and height

A higher proportion of people in the disadvantaged SEP were classified as impaired lung function at any stage of life, trajectories, social mobility, and cumulative score than advantaged SEP (table 2). There was no significant difference between sex, but statistical differences were found for age and smoking status (not shown in tables).

**Table 2:** Impaired lung function by socioeconomic status (numbers and proportions, all countries and both sexes combined, unadjusted)

			Total	Impaired lung function n (%)
Childhood				
Advantaged (0)			3805	622 (16.35)
Disadvantaged (1)			6355	1145 (18.02)
Young adulthood				
Advantaged (0)			3125	410 (13.12)
Disadvantaged (1)			7035	1357 (19.29)
Late adulthood				
Advantaged (0)			5660	914 (16.15)
Disadvantaged (1)			4500	853 (18.96)
Trajectories				
Childhood	Young adulthood	Late adulthood		
0	0	0	975	128 (13.13)
0	0	1	572	89 (15.56)
0	1	0	1111	195 (17.55)
0	1	1	1147	210 (18.31)
1	0	0	1070	131 (12.24)
1	0	1	508	62 (12.20)
1	1	0	2504	460 (18.37)
1	1	1	2273	492 (21.65)
Social mobility				
Always advantaged			975	128 (13.13)
Upward mobility			4082	653 (16.00)
Downward mobility			2830	494 (17.46)
Always disadvantaged			2273	492 (21.65)
Cumulative score				
0, never disadvantaged			975	128 (13.13)
1			2753	415 (15.07)
2			4159	732 (17.60)
3, always disadvantaged			2273	492 (21.65)

The associations between SEP variables and impaired lung function in men and women are shown in tables 3 and 4, respectively. The tables also show the prevalence and age-adjusted ORs of ever-smoking (the most likely confounder or mediator) by SEP. There were social gradients in lung function impairment in both genders which, as might be expected, were most pronounced for models 4 (social mobility) and 5 (disadvantage accumulation), with the age-adjusted ORs (comparing the worst vs best categories) being 1.42 in men and 1.83 in women. Among specific life-course stages (critical period, model 1), the strongest effects of disadvantage were seen for SEP in young adulthood (assessed by education), particularly in women. Factors acting in late adulthood showed modest statistically significant associations in women but not in men. Life course trajectories (model 3) were generally driven by the strengths of associations seen at specific periods.

**Table 3:** Prevalence of ever smoking and ORs for ever smoking and impaired lung function in men

Model type	SEP category	Level 0=advantage 1=disadvantage	Prevalence of ever smoking (%)	OR for ever smoking adjusted for age	OR for impaired lung function	
					Adjusted for age and country	Adjusted for age, country and smoking
Model 1 Critical period	Childhood	0	66.88	1.0	1.0	1.0
		1	66.32	1.06 (0.93–1.21)	1.01 (0.87–1.19)	1.01 (0.86–1.18)
	Young adulthood	0	57.16	1.0	1.0	1.0
		1	70.85	1.82 (1.60–2.07)	1.26 (1.06–1.49)	1.16 (0.97–1.37)
	Late adulthood	0	62.74	1.0	1.0	1.0
		1	72.56	1.52 (1.33–1.73)	1.15 (0.99–1.34)	1.09 (0.94–1.28)
Model 2 Sensitive period	Childhood	0	66.88	1.0	1.0	1.0
		1	66.32	0.95 (0.83–1.08)	0.96 (0.82–1.13)	0.98 (0.83–1.15)
	Young adulthood	0	57.16	1.0	1.0	1.0
		1	70.85	1.77 (1.55–2.02)	1.26 (1.05–1.50)	1.15 (0.97–1.38)
	Late adulthood	0	62.74	1.0	1.0	1.0
		1	72.56	1.43 (1.25–1.63)	1.13 (0.97–1.32)	1.08 (0.93–1.26)
Model 3 Trajectories	Trajectory across three time points	0, 0, 0	55.60	1.0	1.0	1.0
		0, 0, 1	60.08	1.17 (0.86–1.60)	1.13 (0.75–1.71)	1.12 (0.94–1.70)
		0, 1, 0	71.10	1.95 (1.50–2.53)	1.38 (0.99–1.92)	1.25 (0.90–1.75)
		0, 1, 1	77.97	2.71 (2.04–3.61)	1.33 (0.95–1.88)	1.16 (0.82–1.64)
		1, 0, 0	55.30	1.06 (0.82–1.36)	0.97 (0.69–1.38)	0.97 (0.69–1.38)
		1, 0, 1	62.57	1.40 (0.99–1.99)	1.15 (0.73–1.82)	1.10 (0.70–1.74)
		1, 1, 0	65.09	1.58 (1.27–1.95)	1.19 (0.89–1.58)	1.12 (0.84–1.49)
		1, 1, 1	75.45	2.52 (1.99–3.19)	1.43 (1.07–1.92)	1.27 (0.94–1.71)
Model 4 Social mobility	Always up	0, 0, 0	55.60	1.0	1.0	1.0
	Upward mobility	1, 0, 0 1, 0, 1 1, 1, 0	62.28	1.40 (1.15–1.72)	1.13 (0.86–1.49)	1.08 (0.82–1.42)
	Downward mobility	0, 0, 1 0, 1, 0 0, 1, 1	71.37	1.94 (1.56–2.42)	1.31 (0.98–1.74)	1.19 (0.89–1.59)
	Always down	1, 1, 1	75.45	2.53 (2.00–3.20)	1.42 (1.06–1.91)	1.26 (0.94–1.70)
Model 5 Disadvantage accumulation	Number of times disadvantaged, categorical	0	55.60	1.0	1.0	1.0
		1	62.59	1.38 (1.11–1.70)	1.17 (0.88–1.55)	1.12 (0.84–1.49)
		2	67.87	1.77 (1.44–2.17)	1.22 (0.93–1.60)	1.12 (0.85–1.48)
		3	75.45	2.55 (2.02–3.23)	1.42 (1.06–1.91)	1.26 (0.94–1.70)

SEP, socioeconomic position.

**Table 4:** Prevalence of ever smoking and ORs for ever smoking and impaired lung function in women

Model type	SEP category	Level 0=advantage 1=disadvantage	Prevalence of ever smoking (%)	OR for ever smoking adjusted for age	OR for impaired lung function	
					Adjusted for age and country	Adjusted for age, country and smoking
Model 1 Critical period	Childhood	0	45.12	1.0	1.0	1.0
		1	33.06	0.74 (0.65–0.83)	1.15 (0.98–1.35)	1.18 (1.01–1.38)
	Young adulthood	0	31.51	1.0	1.0	1.0
		1	40.21	1.54 (1.36–1.75)	1.56 (1.29–1.88)	1.57 (1.26–1.71)
	Late Adulthood	0	34.04	1.0	1.0	1.0
		1	41.28	1.28 (1.15–1.43)	1.26 (1.09–1.46)	1.25 (1.08–1.45)
Model 2 Sensitive period	Childhood	0	45.12	1.0	1.0	1.0
		1	33.06	0.69 (0.61–0.77)	1.06 (0.90–1.24)	1.08 (0.92–1.28)
	Young adulthood	0	31.51	1.0	1.0	1.0
		1	40.21	1.59 (1.40–1.81)	1.50 (1.23–1.82)	1.50 (1.23–1.82)
	Late adulthood	0	34.04	1.0	1.0	1.0
		1	41.28	1.22 (1.09–1.37)	1.22 (1.05–1.41)	1.20 (1.04–1.40)
Model 3 Trajectories	Trajectory across three time points	0, 0, 0	35.74	1.0	1.0	1.0
		0, 0, 1	43.26	1.22 (0.91–1.64)	1.34 (0.87–2.05)	1.33 (0.87–2.05)
		0, 1, 0	44.27	1.42 (1.11–1.83)	1.35 (0.92–1.96)	1.35 (0.92–1.96)
		0, 1, 1	53.25	1.92 (1.50–2.45)	1.61 (1.13–2.30)	1.57 (1.10–2.25)
		1, 0, 0	24.54	0.69 (0.52–0.90)	0.95 (0.63–1.43)	0.96 (0.63–1.45)
		1, 0, 1	25.23	0.69 (0.50–0.94)	0.93 (0.57–1.50)	0.94 (0.58–1.52)
		1, 1, 0	32.67	1.06 (0.85–1.33)	1.49 (1.07–2.07)	1.53 (1.10–2.13)
		1, 1, 1	38.56	1.30 (1.04–1.62)	1.87 (1.35–2.58)	1.89 (1.37–2.61)
Model 4 Social mobility	Always up	0, 0, 0	35.74	1.0	1.0	1.0
	Upward mobility	1, 0, 0 1, 0, 1 1, 1, 0	29.40	0.89 (0.72–1.10)	1.28 (0.93–1.75)	1.30 (0.96–1.79)
	Downward mobility	0, 0, 1 0, 1, 0 0, 1, 1	47.96	1.57 (1.27–1.95)	1.42 (1.02–1.96)	1.40 (1.01–1.94)
	Always down	1, 1, 1	38.56	1.30 (1.04–1.62)	1.83 (1.32–2.52)	1.84 (1.33–2.55)
Model 5 Disadvantage accumulation	Number of times disadvantaged, categorical	0	35.74	1.0	1.0	1.0
		1	36.65	1.08 (0.86–1.34)	1.19 (0.86–1.66)	1.20 (0.86–1.67)
		2	38.01	1.24 (1.00–1.53)	1.43 (1.04–1.95)	1.44 (1.05–1.97)
		3	38.56	1.32 (1.06–1.65)	1.83 (1.32–2.52)	1.85 (1.34–2.55)

The prevalence and social gradient in ever-smoking (also shown in tables 3 and 4) were considerably more pronounced in men than in women. Consistently with the prevalence pattern, the adjustment for smoking substantially attenuated the ORs in men (eg, from 1.42 to 1.26 for the worst vs best categories of disadvantage accumulation), while it did not materially change the ORs in women.

Country-specific ORs are shown in online supplemental table 1 (adjusted for age and sex). For all SEP indicators, the associations were consistently strongest in the Lithuanian cohort, compared with the other two cohorts. However, the heterogeneity by country was not statistically significant (all p values >0.12). All countries tended to follow a similar pattern, whereas young adulthood disadvantaged SEP and being disadvantaged at all three stages of life had significant negative effects on pulmonary health.

10.1136/jech-2022-219348.supp3 Supplementary data

## Discussion

By using data from 10 160 individuals from three Central and Eastern European countries, we showed that disadvantaged SEP in young and late adulthood was associated with higher odds of impaired lung function, while the associations with childhood SEP were not significant. These results confirm that SEP measured at different stages of life is a good predictor of pulmonary health and can predict impaired lung function. Smoking, particularly among men, made a substantial contribution to the social differences. The results were broadly consistent between the three cohorts, suggesting that focusing on only one period of life can lead to an incomplete assessment of social inequalities.

Several studies have used life-course SEP indicators to predict the mean value of FEV_1_ and FVC at different stages of life.^1 2 12 28 29^ All of them reported that disadvantaged SEP was associated with reduced lung function from 100 mL up to 310 mL in men and women. As a difference of 100 mL in FEV_1_ has been recognised as the minimal clinically important difference by the ATS/ERS when pulmonary drugs are used,^30 31^ the observed reductions in FEV_1_ by disadvantaged SEP may have clinical implications. With our results, we were able to demonstrate that disadvantaged SEP is associated not only with the reduction of lung function but also with impaired lung function classified using clinical criteria.

SEP could impact lung function from childhood to adulthood. Our analysis used maternal education as a proxy for childhood SEP, although some studies suggest that the association between parents’ education and children’s health may be weaker than expected.^32^ Longitudinal studies have shown that children living in disadvantaged social conditions have a reduced FEV_1_ and FVC in later stages of life compared with those in middle and high SEP.^12 28 29 33^ Childhood disadvantaged SEP has been reported as having a detrimental effect on prenatal and postnatal lung growth and development as maternal smoking, preterm birth and postnatal pulmonary infections are linked with poverty; the consequences of these factors have been observed to persist in adult life.^34^ In our data, we did not observe statistically significant associations between childhood disadvantaged SEP and the risk of impaired lung function, and we observed that children with upward social mobility were not at significantly increased risk of impaired lung function. However, we remain cautious about this result, because the classification of SEP indicators varies from author depending on the availability of data^13^; it is possible that maternal education in these CEE studies does not adequately capture childhood SEP.

Different mechanisms can affect pulmonary health at different life stages. Alveoli production stops at the age of 7, and after that, lung capacity and volumes are dependent mainly on thorax enlargement after that age.^35^ Some authors described adolescence as a physiological period of alveolarisation and thus vulnerability to injuries.^36 37^ It could help understand that our results suggest that the strongest effect of disadvantaged SEP was in young adulthood for critical and sensitive periods. Around the age of 20, pulmonary capacity reaches a plateau and decreases slowly with age, but exposition to pulmonary risk factors such as high air pollution or overcrowding condition can accelerate the loss of lung capacity and increase mortality from chronic pulmonary diseases.^38^ These pulmonary risk factors are more commonly observed in disadvantaged socioeconomic groups.^39–41^ Our findings strongly suggest an important effect of smoking on the social gradient in lung function impairment. In these CEE studies, smoking was considerably more common in men, and, consistently, adjustment for smoking led to a substantial attenuation of the ORs in men but not in women. This observation suggests that smoking may partly mediate the association between SEP and lung functions in men. On the other hand, the social gradient in lung function in women appears to be driven by factors other than smoking.

It has been proposed that people who transitioned to higher SEP have higher FEV_1_ and FVC than those who moved to lower SEP.^2 12^ This is consistent with our observation that those with upward mobility are not at higher odds of impaired lung function than those always advantaged; in contrast, downward mobility was associated with higher odds of impaired lung function. We observed that individuals disadvantaged at three points in time had the highest odds of impaired lung function. These subjects did not have the opportunity to recover from the damage at any time point nor to start their lives with normal pulmonary development. Thus, the longer a person lived in a disadvantaged SEP, the higher the odds of impaired lung function.

### Strengths and limitations

An important strength of the study is the scheme used to classify lung functions. In most epidemiological studies, when disadvantaged SEP was used to predict FEV_1_ and FVC decline, a new linear regression model was built. Instead, Rocha *et al* 2 proposed that standardised equations should be used to calculate the predicted values. Further, we classified whether a person had impaired lung function. This approach also helped us to systematically analyse if FEV_1_ reduction up to >300 mL in men and >200 mL in women described by Hagewald and Crapo^1^ was the consequence of the different impact of SEP by sex or if these trends might be explained by physiological differences.^42^ According to our results, the interaction between sex and disadvantage accumulation was not significant; in fact, sex was not significant in any of the models shown here. Finally, by using ORs, we were able to demonstrate that previously described loss of lung function in disadvantaged SEP has a clinical impact.

This study also has several limitations. First, while respiratory symptoms are important for accurate diagnosis of impaired lung function, the presence of symptoms in absence of spirometry abnormal findings needs extra pulmonary tests such as diffusion capacity of carbon monoxide and total lung capacity, which were not available. Consequently, a potential overdiagnosis of impaired lung function should be considered here. Furthermore, spirometry tests were done only once, with a longitudinal analysis of pulmonary health being unfeasible to conduct.

Second, the measures of SEP available in our study may be subject to misclassification and reporting bias, and they do not capture the full range of all possible SEP indicators determining health. The lack of association between lung function and childhood SEP may be due to simplistic measurement and lack of variability in maternal education. In preliminary analyses, the father’s education was also considered, but it had a slightly higher number of missing values (227 individuals) and produced similar results as maternal education.

Third, we do not have prospective data over the life course about SEP and other respiratory risk factors in childhood (eg, preterm delivery or passive smoking) or later (eg, smoking trajectories). The cross-sectional data do not allow establishment of temporality and reliably identify mediators. Further research is needed to clarify the interaction between SEP and trajectories in smoking, physical activity and other factors. For example, SEP is not the only driver of smoking.^43^ Similarly, people with pulmonary impairment have lower levels of physical activity, but it is often unclear what comes first.^44 45^ For future research, it is important to capture these variables, ideally in large birth cohorts.

## Conclusion

This study shows that models that used three time points concerning SEP performed better than single time points. Disadvantaged SEP was associated with higher odds of impaired lung function in life-course analysis. Reducing socioeconomic inequalities would likely contribute to reducing the risk of impaired lung function in adults.

## Data Availability

Data are available upon reasonable request.
