# Supplementary figures and images for: Socioeconomic position over the life course and impaired lung function of older adults in Central and Eastern Europe: the HAPIEE study
Source: J Epidemiol Community Health. 2022 Nov 2;77(1):49–55. doi: 10.1136/jech-2022-219348 (PMC9763222; doi:10.1136/jech-2022-219348)

Supplementary Figure 1

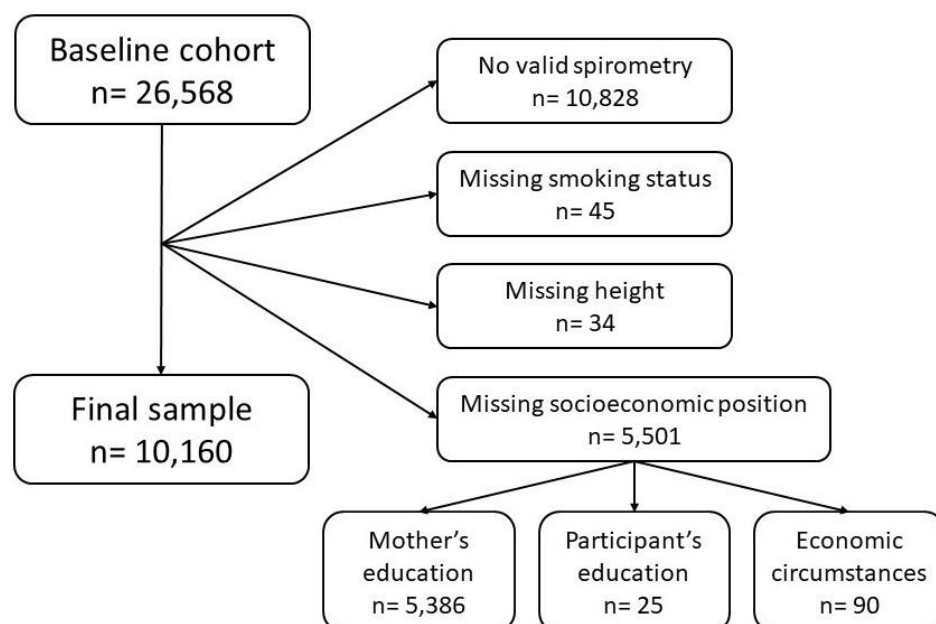

Supplement: Supplementary data [file jech-2022-219348supp001.pdf]
