# Supplementary material for: Socioeconomic position over the life course and impaired lung function of older adults in Central and Eastern Europe: the HAPIEE study
Source: J Epidemiol Community Health. 2022 Nov 2;77(1):49–55. doi: 10.1136/jech-2022-219348 (PMC9763222; doi:10.1136/jech-2022-219348)
Supplement: Supplementary data [file jech-2022-219348supp002.pdf]

Supplementary Figure 2

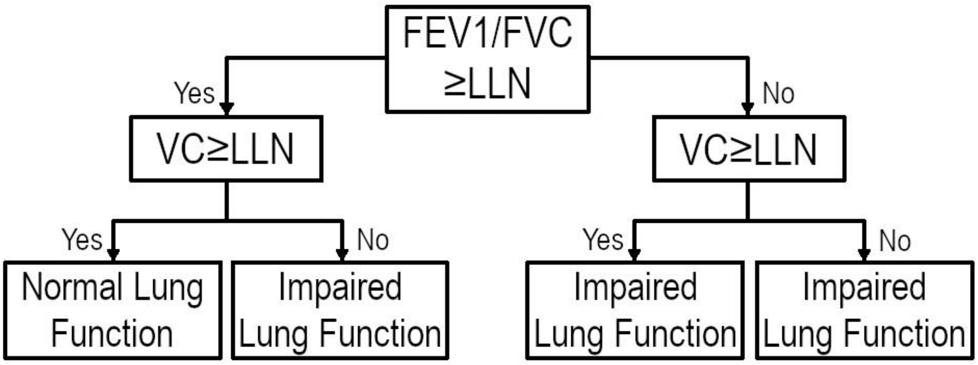

FEV1/FVC Forced expiratory volume divided by forced vital capacity
VC Vital capacity
LLN Lower limit of normality
