# Supplementary material for: Socioeconomic position over the life course and impaired lung function of older adults in Central and Eastern Europe: the HAPIEE study
Source: J Epidemiol Community Health. 2022 Nov 2;77(1):49–55. doi: 10.1136/jech-2022-219348 (PMC9763222; doi:10.1136/jech-2022-219348)
Supplement: Supplementary data [file jech-2022-219348supp003.pdf]

Supplementary Table 1. Odds ratio (95% CI) for impaired lung function by SEP indicators adjusted for age and sex.

Model type	SEP category	Level 0=Adv 1=Disadv	Czech Republic n= 3,620	Poland n= 3,871	Lithuania n= 2,669
**MODEL 1 Critical period**	Childhood	0	1.0	1.0	1.0
		1	1.06 (0.88–1.27)	1.09 (0.92–1.28)	1.11 (0.83–1.49)
	Young Adulthood	0	1.0	1.0	1.0
		1	1.33 (1.02–1.72)	1.38 (1.15–1.65)	1.45 (1.13–1.85)
	Late Adulthood	0	1.0	1.0	1.0
		1	1.09 (0.92–1.30)	1.19 (1.02–1.39)	1.53 (1.19–1.98)
**MODEL 2 Sensitive period**	Childhood	0	1.0	1.0	1.0
		1	1.03 (0.85–1.23)	1.00 (0.84–1.19)	1.02 (0.76–1.38)
	Young Adulthood	0	1.0	1.0	1.0
		1	1.31 (1.00–1.70)	1.35 (1.12–1.63)	1.42 (1.10–1.82)
	Late Adulthood	0	1.0	1.0	1.0
		1	1.07 (0.90–1.28)	1.16 (0.99–1.35)	1.51 (1.17–1.94)
**MODEL 3 Trajectories**	Trajectory across three-time points	0, 0, 0	1.0	1.0	1.0
		0, 0 ,1	1.11 (0.60–2.06)	1.15 (0.76–1.73)	1.49 (0.82–2.70)
		0, 1, 0	1.44 (0.94–2.21)	1.27 (0.88–1.85)	0.78 (0.36–1.70)
		0, 1, 1	1.31 (0.85–2.01)	1.49 (1.04–2.12)	2.07 (0.98–4.36)
		1, 0, 0	1.24 (0.67–2.29)	0.88 (0.59–1.32)	0.99 (0.63–1.58)
		1, 0, 1	0.76 (0.32–1.81)	1.17 (0.72–1.92)	1.02 (0.59–1.76)
		1, 1, 0	1.25 (1.83–1.90)	1.33 (0.98–1.81)	1.27 (0.81–1.99)
		1, 1, 1	1.56 (1.04–2.35)	1.51 (1.11–2.04)	2.25 (1.41–3.60)
**Model 4 Social mobility**	Always up	0, 0, 0	1.0	1.0	1.0
	Upward mobility	1, 0, 0<br>1, 0, 1<br>1, 1, 0	1.22 (0.81–1.83)	1.22 (0.91–1.64)	1.11 (0.74–1.67)
	Downward mobility	0, 0, 1<br>0, 1, 0<br>0, 1 ,1	1.35 (0.90–2.01)	1.32 (0.97–1.80)	1.32 (0.81–2.17)
	Always down	1, 1, 1	1.56 (1.03–2.35)	1.51 (1.11–2.04)	2.25 (1.41–3.59)
**MODEL 5 Disadvantage accumulation**	No. times disadvantaged, categorical	0	1.0	1.0	1.0
		1	1.36 (0.90–2.06)	1.10 (0.80–1.51)	1.05 (0.68–1.62)
		2	1.25 (0.84–1.86)	1.36 (1.01–1.82)	1.26 (0.82–1.92)
		3	1.56 (1.03–2.35)	1.51 (1.11–2.05)	2.28 (1.43–3.64)

SEP Socioeconomic position; *Adv* Advantaged SEP; *Disadv* Disadvantaged SEP
